# The Role of Toll Like Receptors in Pregnancy

**Published:** 2013-09-18

**Authors:** Elham Amirchaghmaghi, Seyed Abdolvahab Taghavi, Farnaz Shapouri, Shaghayegh Saeidi, Abbas Rezaei, Reza Aflatoonian

**Affiliations:** 1 Department of Immunology, Faculty of Medicine, Isfahan University of Medical Sciences, Isfahan, Iran; 2 Department of Anatomy, School of Medicine, Iran University of Medical Sciences, Tehran, Iran; 3 Department of Endocrinology and Female Infertility at Reproductive Biomedicine Research Center, Royan Institute for Reproductive Biomedicine, ACECR, Tehran, Iran

**Keywords:** Innate Immunity, TLRs, Pregnancy, PRRs

## Abstract

For many years, the innate immunity was of less interest than the adaptive immunity because it was perceived to have secondary importance in the functionality of the immune system. During the past decades, with the advancement of knowledge about innate immune system, interest in innate immunity has grown dramatically and thus its function has been extensively studied. Innate immunity plays fundamental roles in the initiation and induction of adaptive immune responses. It consists of several cells and receptors including natural killer (NK) cells, macrophages (MQs), dendritic cells (DCs) and pattern recognition receptors (PRRs). Two decades ago, Toll like receptors (TLRs) family was known as one of the important PRRs with unique functions especially in protection against invading pathogens. Since the female reproductive tract has access to the outside environment and has a unique interaction with different pathogens whether invading microorganisms or normal flora, allogenic sperm and semi allogenic fetus, it has an essential need for effective immune responses. It has therefore been suggested that TLRs may play important roles in the immune regulation of the female reproductive tract. In addition, it has been demonstrated that immune disturbance may be responsible for some adverse pregnancy outcomes such as preeclampsia (PE), recurrent spontaneous abortion (RSA) and intrauterine growth restriction (IUGR). Our focus in this review is to show the importance of TLRs in pregnancy with emphasis on the expression of these receptors in different tissues related to pregnancy.

## Introduction

All organisms are in a continuous challenge with the surrounding environment during their life. The defense mechanism of every organism which is called the immune system has two main branches in vertebrates. Innate (natural) immunity, which is the first branch of immune system, is the ancient form of host defense against infection and plays a critical role in activation of adaptive (acquired) immunity, the another branch of the immune system. The adaptive immune system responds to specific 'non-self ' antigens and generates immunologic memory. Innate immunity comprises different cells and receptors which provides first line of defense against invading microorganisms (1). Pathogens that invade a vertebrate host are initially recognized by the innate immune system via a limited number of germline-encoded receptors called patternrecognition receptors (PRRs) (1, 2).

PRRs have some common characteristics including: 1. They are able to recognize different microbial components known as pathogen associated molecular patterns (PAMPs). PAMPs are essential for the survival of the microorganism and therefore it could not alter them without threatening its life. 2. Their expression is constitutive in the host and thus could detect the pathogens during their life time. 3. PRRs are germline encoded and nonclonal which are expressed on all cells of a specific type (2, 3).

PRRs not only recognize exogenous components derived from both pathogenic and non pathogenic microorganisms known as PAMPs, but also respond to endogenous molecules released from dying host cells upon cellular stress or tissue injury known as damage associated molecular patterns (DAMPs) (4, 5).

PRRs exist in every compartment of the body. Some of them are humoral proteins circulating in the plasma while endocytic receptors expressed on the cell surface and signaling receptors can be expressed either on the cell surface or intracellularly (3). The PRRs come under two types of Toll like receptors (TLRs) and nod-like receptors (NLRs) as membranous and intracellular receptors, respectively (6).

### Toll like receptors

One of the main subgroups of PRRs which are conserved during evolution is TLRs. They are type I transmembrane glycoproteins which consist of extracellular domains containing varying numbers of leucine-rich-repeat (LRR) motifs, a trans membrane portion and a cytoplasmic signaling domain homologous to that of the interleukin-1 receptor (IL-1R), termed the Toll/ IL-1R homology (TIR) domain (7).

To date, different TLRs have been identified in different species. TLRs 1-9 are conserved between human and mouse. Although TLR10 is not functional in mice, they express TLR11, TLR12 and TLR13 which are not expressed in humans (8).

In a host, TLRs are expressed in various cells. They are not only expressed in immune cells such as macrophages (MQ), dendritic cells (DCs), B lymphocytes and specific types of T cells but are also expressed in non-immune cells including fibroblasts and epithelial cells. In addition, expression of TLRs is dynamic and rapidly changes in response to pathogens, a variety of cytokines and environmental stresses (2).

### History of TLRs

TLRs are named because of their similarity to a molecule identified in the fruit fly, Drosophila melanogaster called 'Toll'. Toll was first identified by Anderson et al. in 1985 as a gene in Drosophila which its protein product plays an important role during its embryogenesis in development of dorsal-ventral axis (9). In 1996, Lemaitre et al. (10) revealed that Toll protein had an important role in the immune response of the fly against fungal infection by inducing antifungal peptide expression.

Later, receptors were identified which were similar to Toll so were named "Toll like receptors". The first human TLR was reported by Nomura et al. in 1994 (11) and was mapped on chromosome 4 by Taguchi et al. in 1996 (12). At that time, it was assumed that TLRs are important in the developmental process. In 1997, Charles Janeway and Ruslan Medzhitov showed that activation of a TLR could induce the activation of certain genes necessary for initiating an adaptive immune response. They cloned and characterized a human homologue of the Drosophila Toll protein and showed that like Toll in Drosophila, human Toll is a type I transmembrane protein with extracellular and cytoplasmic domains. They showed that activation of this protein can induce the activation of the transcription factor, nuclear factor -kappa B (NFkB). Subsequently, it induces the expression of NF-kB-controlled genes including the inflammatory cytokines IL-1, IL-6 and IL-8 as well as the expression of B7.1. As a co-stimulatory molecule, B7.1 is required for the activation of naive T cells (1, 3). Of note, the first identified human TLR is now known as TLR4.

### TLRs classification

**Fig 1:**
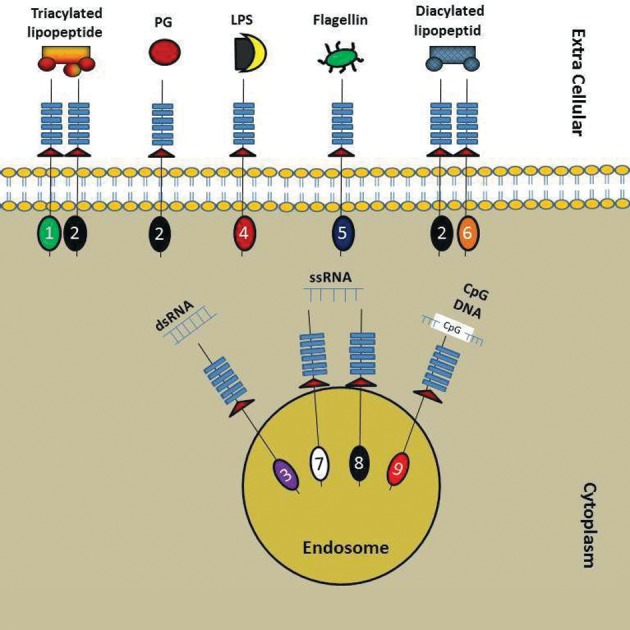
Distribution and dimerisation of TLRs in different cellular compartments (2, 8). PG; Peptidoglycan, LPS; Lipopolysaccharide, dsRNA; Double stranded RNA and ssRNA; Single stranded RNA.

TLRs are classified according to their cellular localization and respective PAMP ligands. Cell surface TLRs (TLR1, TLR2, TLR4, TLR5, TLR6 and TLR10) are expressed on the cell membrane and recognize mainly microbial membrane components such as lipids, proteins and lipoproteins. On the other hand, TLR3, TLR7, TLR8 and TLR9 are exclusively expressed on intracellular compartments such as the endoplasmic reticulum (ER), endosomes, lysosomes and endolysosomes and recognize nucleic acids including single and double stranded RNA (ssRNA , dsRNA ) and DNA (2, 8) (Fig 1).

### TLRs signaling

Although extracellular domain of each TLR (corresponding to ligand recognition) is different from others, all of them have a great similarity in their intracellular domain and respective transduction pathways.

**Fig 2:**
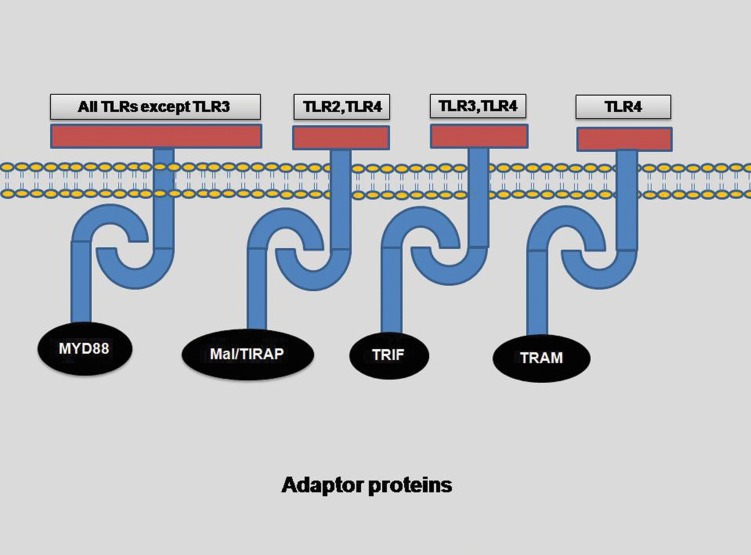
Adaptor molecules involved in different TLRs signaling (2, 13).

After ligand binding, the intracellular cascades start. As mentioned previously, all TLRs have cytoplasmic signaling domain homologous to that of IL-1 receptor, known as TIR domain. Several adaptor molecules containing TIR domain interact with the TIR domain of TLRs. These adaptors include myeloid differentiation primary response gene -88 (MyD88), TIR domain containing adaptor protein/ MyD88 adapter-like protein (TIRAP/Mal), TIR-domain-containing adapter-inducing interferon-β (TRIF) and TRIF-related adaptor molecule (TRAM) (Fig 2). Overall, the downstream signaling pathways of TLRs can be divided into two main groups: MyD88 dependent and MyD88 independent (TRIF dependent) pathways. All TLRs except TLR3 use MyD88 dependent pathway whereas TLR4 could utilize both pathways (2). In addition, TLR4 is the only TLR which recruits all 4 adaptor molecules (MyD88, TIRAP, TRIF and TRAM) for signaling (13). In brief, TLRs 5, 7, 8 and 9, in contrast to TLR3 which uses TRIF, use only MyD88 while TLR2 in dimer form with TLR1 or TLR6 recruits both MyD88 and TIRAP (13). TLRs activation could result in:

- production of inflammatory cytokines and chemokines.
- induction of anti viral response by production of type 1 interferons.
- maturation of dendritic cells by upregulation of costimulatory molecules (13).

### MyD88 dependent pathway

Following the engagement of adaptor molecules, different molecules are recruited including several IL-1 receptor associated kinases (IRAKs) and TNF receptor associated factors (TRAFs) and mitogen activated protein kinases (MAPKs). Afterwards, inhibitory kappa B kinase (IKK) is engaged and modulates the activation and translocation of the transcription factor NF-κB. Another transcription factor activated by MAPKs is activating protein-1 (AP-1) (13). In case of TLRs 7, 8 and 9, interferon response factor 7 (IRF7), a transcription factor, is activated and translocated to the nucleus. Finally, this pathway leads to the production of inflammatory cytokines and/or type 1 interferons (IFN I) (2) depending on which TLR was activated.

### MyD88 independent (TRIF dependent) pathway

**Fig 3:**
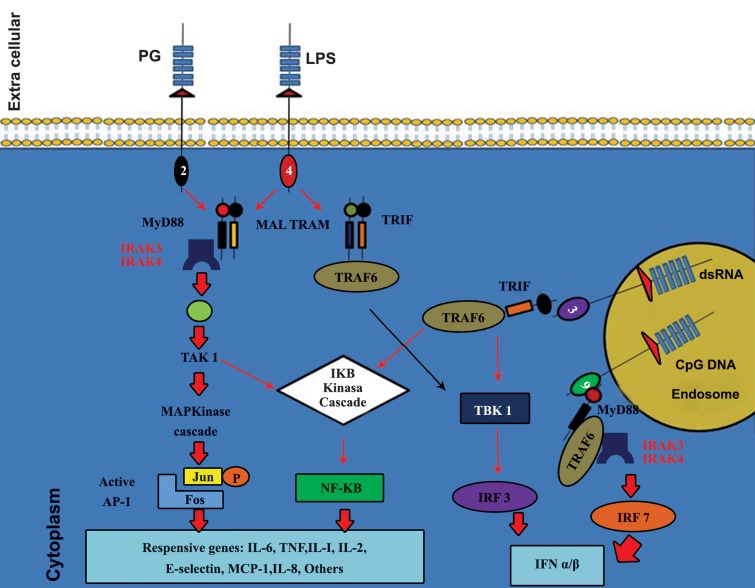
Signaling pathways of different TLRs (2).

Both TLR3 and TLR4 use this pathway which leads to the recruitment of TRAFs and IRF3 and production of both inflammatory cytokines and IFN I (2) (Fig 3).

In particular, anti viral responses are induced by TLRs 3 and 4 activation (using TRIF) or TLRs 7, 8 and 9 activation (using MyD88 pathway).

### TLRs ligands

Each TLR has its own distinct PAMP ligands. The cell surface TLRs recognize different ligands including components of gram positive and gram negative bacteria, fungi and parasites such as lipopolysaccharide (LPS), peptidoglycan (PG), lipoteichoic acid (LT), flagellin, mannan, zymosan and glycoinositolphospholipids. On the other hand, intracellular TLRs are usually stimulated by nucleic acids of viruses and bacteria including ssRNA, dsRNA and CpG unmethylated DNA (Table 1) (2, 14-18). Recently, some host derived molecules are identified as endogenous ligands for TLRs such as some heat shock proteins (HSP) 60 and 70 (19), neutrophil elastase (20), fatty acid, heme (21), beta defensin (22), reactive oxygen species (ROS) (23), fibronectin (24), oligosaccharides of hyaluronic acid, heparan sulfate (25) and chromatin-IgG complexes (26).

As mentioned above, despite relatively limited types of TLRs known in human (TLR1-10), they can react with a wide spectrum of PAMPs. This could be explained by a special characteristic of TLRs. TLRs usually form dimers (homo or hetero dimers) which increases the diversity of ligands recognized by them, for example, TLR2 usually forms heterodimers with TLR1 or TLR6 with each dimer having different ligand specificity (27, 28). The varieties of ligands recognized by TLRs underline the importance of the TLRs in the host innate immune response.

**Table 1:** Human TLRs, their respective ligands (2, 14-19, 26)


TLRs	Ligands

**TLR1**	Triacyl lipopeptides
**TLR2**	Diacyl lipopeptides, Triacyl lipopeptides, Lipoteichoic acid, Peptidoglycans, Porins, Lipoarabinomannan, Phospholipomannan, Zymosan, Hemagglutinin protein
**TLR3**	Double stranded RNA
**TLR4**	LPS, Mannan, Heat-shock protein 60, 70, Fibrinogen
**TLR5**	Flagellin
**TLR6**	Diacyl lipopeptides, Lipoteichoic acid, Zymosan
**TLR7**	ssRNA
**TLR8**	ssRNA
**TLR9**	CpG-DNA, Chromatin-IgG complex
**TLR10**	Unknown


### TLRs and female reproductive tract

Several reports have revealed that TLRs are expressed throughout different parts of the female reproductive tract (29, 30). In addition, it was shown that their expression is altered during different phases of the menstrual cycle (31), suggesting that alterations during the menstrual cycle may be under the control of sex hormones including estrogen and progesterone (31, 32). During pregnancy, special hormonal changes and immunologic challenges occur. With regard to the immune system, normal pregnancy consists of three different immunologic phases:

1. Pro-inflammatory environment during embryo implantation, placentation and early stage of pregnancy.
2. Anti-inflammatory milieu during mid-pregnancy
3. Pro-inflammatory environment at third trimester and end of pregnancy (33).

During normal pregnancy, different parts of female reproductive tract including endometrium, myometrium, cervix and vagina undergo histological and functional changes while specific pregnancy related tissues such as amnion, chorion and placenta are created.

In the following, we will overview the researches done on the expression of different TLRs and their function during pregnancy in different tissues of the female reproductive tract which are closely in relation to embryo.

### Placenta

The placenta serves as an active barrier between the embryo and the surrounding environment. Different PRRs are considered to play roles in this interaction including TLRs and NLRs (34).

Various studies have evaluated the presence and function of TLRs or their related molecules in placental tissues. By using immunohistochemistry, Kumazaki et al. investigated the expression of TLR4 protein in human placentas obtained from normal and complicated pregnancies delivered in the second and third trimesters. They showed that TLR4 was found on the extravillous trophoblasts, intermediate trophoblasts/X cells in the degenerative villi, and villous Hofbauer cells of both preterm and term placentas and on the inflammatory cells in placentas with chorioamnionitis (CAM). TLR4 immunoreactivity was increased in the villous Hofbauer cells of preterm CAM placentas compared with those of preterm placentas without CAM or those of term placentas with or without CAM (35). Later, Abrahams et al. studied the expression and function of TLR2 and TLR4 in first trimester trophoblast cells. They found that activation of TLR4 induces cytokine production by trophoblast cells, but TLR2 activation induced apoptosis using Fas associated death domain, the inactivation of the X-linked inhibitor of apoptosis, and the activation of caspases 3, 8 and 9. They suggested a pathogenic role for TLR2 during some intrauterine infections (36).

Klaffenbach et al. studied the chorioncarcimoma cell lines by using different techniques including real time polymerase chain reaction (real time PCR), fluorescence-activated cell sorter analysis and immunoblat. They showed that LPS and CpG DNA increases the expression of TLR2 mRNA and protein (37). Nishimura and Naito studied different fetal and adult tissues including placenta by real time PCR and showed the mRNA of all ten human TLRs was expressed while TLR3 mRNA was expressed at the highest level in the placenta in comparison with other tissues (38). Patni et al. compared human term placentas collected during elective caesarean sections (ECS) with those of normal vaginal delivery (NVD) to explore the effect of completion of labor on TLR expression profile. They showed that both groups of placentas expressed the TLR1-TLR10 and revealed for the first time that human term placenta can respond to TLR3, TLR5 and TLR7/8 agonists (39).

Activation of TLRs on trophoblast has various consequences during pregnancy including immune cell recruitment, cytokine secretion and protective responses to invading pathogens (40). Recently, Aboussahoud et al. used an *in vitro* model of human embryo implantation and showed that activation of TLR5 decreases the attachment of human trophoblast cells to endometrial cell line (41). In addition, it has been suggested that pregnancy complications associated with placental dysfunction, such as preterm labor, may be a result of TLR activation (40). It seems that proper interaction between TLRs and respective ligands on placenta plays important roles during different phases of pregnancy including implantation and labor.

### Chorion and amnion

The expression of TLRs in fetal membrane such as amnion has been studied but not as extensive as placenta. Kim et al. showed that the expression of TLR2 and TLR4 is increased in chorioamnion membrane at time of labor and in presence of chorioamnionitis. They also showed that TLR2 was polarized to the basal surface of amniotic epithelial cells in women without inflammation but this distribution was lost in the presence of chorioamnionitis (42). Recently, Choi et al. studied the immunohistochemical expression of TLR4 in different histological layers and anatomical regions of human fetal membranes. They showed that chorion expressed significantly higher levels of TLR4 than the amnion and this expression did not differ with regard to anatomical regions (uterine fundus vs. uterine low segment). Furthermore, histological presence of chorioamnionitis did not alter TLR4 expression while the progression of gestation significantly decreased the TLR4 expression (43).

### Myometrium

Little data exists in regard to TLRs expression in human myometrium. Youssef et al. demonstrated that expression of TLRs 2 and 4 was significantly higher in pregnant myometrium at term in comparison with preterm. In addition, they showed that the level of TLR2 protein significantly increased during labor. The authors suggest that these TLRs may be important in labor and their function could be suppressed by progesterone (44). 

### Endometrium

For the first time, Young et al. showed that TLRs 1-6 and 9 are expressed in both whole endometrium and separated endometrial epithelial cells using reverse transcriptase-PCR (RT-PCR). They also showed that Ishikawa cells expressed TLRs 2 and 5 while RL95-2 cells expressed TLRs 3, 5 and 9 (29). Subsequently, several studies were undertaken in this regard using different techniques. Pioli et al. reported the expression of TLRs 1 to 6, MyD88 and CD14 in different parts of the female reproductive tract including uterine endometrium (45). Fazeli et al. (30) showed that TLRs 1, 2, 3, 5 and 6 proteins were present in different parts of female reproductive tract. Also they found that TLR4 was only present in upper parts of the reproductive tract including the endocervix, endometrium and fallopian tubes and absent in vagina and ectocervix. 

Aflatoonian et al. studied endometrium obtained from normal women at different phases of menstrual cycle (menstruation, proliferative and secretory) and detected TLRs 7-10 proteins in both endometrial epithelium and stroma. The authors also demonstrated that all ten TLRs were expressed in human endometrial tissue and most of them had significantly higher expression during the secretory phase in comparison with other phases of the menstrual cycle (31). Their findings support that TLRs expression may be under the control of female sex hormones (estrogen and progesterone). It seems that estrogen has an inhibitory effect on TLRs expression while progesterone may have stimulating effects because TLRs expression was at its highest in secretory phase (31, 32). 

 expression in endometrium were not limited to in vivo studies. Aboussahoud et al. investigated TLRs expression and function in three human endometrial epithelial cell lines. They revealed that these established cell lines not only express TLRs but also respond to their known agonists and could be used as reliable *in vitro* models of human endometrium (46).

### Decidua

Decidua is defined as the transformed endometrium during pregnancy, which forms the maternal part of the placenta. For the first time , Krikun et al. studied the decidual tissues and cells obtained from women undergoing first trimester elective terminations or repeat cesarean sections and showed that human decidua differentially express TLRs (47). Canavan and Simhan showed that decidual cells from term unlabored pregnancies express TLRs 1, 2, 4 and 6 which respond to LPS and PG stimulation (48). Recently, Hayati et al. studied TLRs 2 and 4 expression in decidua and amniotic cells of non inflamed placenta and placenta with infection. They reported higher expression of TLR2 in the amniotic and decidua cells of inflamed placenta than non-inflamed placenta, supporting the potential role of TLR2 in defense against infection (49). In addition, Schatz et al. studied the immunostaining of TLR4 protein in decidual cells compared with trophoblasts in different trimesters and showed that TLR4 expression is higher in decidual cells than trophoblasts. They suggested that decidual cells are primary targets for gram negative bacterial infection (50). 

## Conclusion

Pregnancy is a fundamental stage in life of every woman in reproductive age. The immunologic features of normal pregnancy are unique because the mother tolerates the semi allogenic embryo. The immunologic changes during pregnancy are very important not only in normal tissues of the female reproductive tract but also in embryo-related tissues created during pregnancy such as fetal membrane and placenta. According to different findings obtained using several study models including *in vitro* models, animal models or studies done on tissues obtained during normal or complicated human pregnancies, it seems that the TLRs family as one of the main regulators of the innate immunity, is not only involved in protecting the female reproductive tract against invading pathogens, but also is a key regulator in immunologic events during stages of normal pregnancy such as implantation or labor. One of the issues that make researches about TLRs so interesting is the accessibility to the agonists and antagonists of TLRs and the possibility to stimulate or suppress TLRs function. Although researches in regard to roles of TLRs in pregnancy are in progression, more studies are needed especially in cases of pregnancy complications such as preeclampsia, abortion and preterm labor.
